# Phytochemicals as Innovative Therapeutic Tools against Cancer Stem Cells

**DOI:** 10.3390/ijms160715727

**Published:** 2015-07-10

**Authors:** Emanuele-Salvatore Scarpa, Paolino Ninfali

**Affiliations:** Department of Biomolecular Sciences, University of Urbino Carlo Bo, Urbino (PU) 61029, Italy; E-Mail: emanuele.scarpa@uniurb.it

**Keywords:** cancer stem cells, chemoprevention, herbal extracts, phytochemicals, therapeutic agents, molecular mechanisms, self-renewal, metastases

## Abstract

The theory that several carcinogenetic processes are initiated and sustained by cancer stem cells (CSCs) has been validated, and specific methods to identify the CSCs in the entire population of cancer cells have also proven to be effective. This review aims to provide an overview of recently acquired scientific knowledge regarding phytochemicals and herbal extracts, which have been shown to be able to target and kill CSCs. Many genes and proteins that sustain the CSCs’ self-renewal capacity and drug resistance have been described and applications of phytochemicals able to interfere with these signaling systems have been shown to be operatively efficient both *in vitro* and *in vivo*. Identification of specific surface antigens, mammosphere formation assays, serial colony-forming unit assays, xenograft transplantation and label-retention assays coupled with Aldehyde dehydrogenase 1 (ALDH1) activity evaluation are the most frequently used techniques for measuring phytochemical efficiency in killing CSCs. Moreover, it has been demonstrated that EGCG, curcumin, piperine, sulforaphane, β-carotene, genistein and the whole extract of some plants are able to kill CSCs. Most of these phytochemicals act by interfering with the canonical Wnt (β-catenin/T cell factor-lymphoid enhancer factor (TCF-LEF)) pathway implicated in the pathogenesis of several cancers. Therefore, the use of phytochemicals may be a true therapeutic strategy for eradicating cancer through the elimination of CSCs.

## 1. Introduction

Our approach to cancer treatment has been based on the idea that tumors undergo a series of genetic mutations, which result in the activation or overexpression of genes promoting proliferation (oncogenes), the silencing of tumor suppressor genes and development of cancer cells’ ability of cancer cells to elude apoptosis [1]. This leads to the unchecked growth of the tumor and its progression to metastases [2]. However, this traditional concept of tumor growth was called into question when researchers became aware of the existence of cancer stem cells (CSCs) [3,4]. CSCs are cells within a tumor that possess the capacity to self-renew and to originate the heterogenous lineages of cancer cells that comprise the tumor. CSCs may arise from tissue-specific stem cells (SCs) by mutation of genes that make the stem cell cancerous, but it is also conceivable that more differentiated cells can, through multiple mutagenic events, acquire the self-renewal capacity and immortality that typify CSCs [5]. Both tissue-specific SCs and CSCs may account for only a small fraction of cells in any given tissue or tumor, but they are able to maintain their numbers through a typical combination of symmetric and asymmetric divisions [6]: the former produces two SC daughters, the latter one SC and one non-SC daughter. The underlying mechanisms in generating excess of CSCs (e.g., in tumor development) are related to an increase in symmetric division [5,7].

Current chemotherapeutic agents and radiation therapy largely target proliferating and differentiated cells, which form the bulk of the tumor, but not CSCs, which are presumably arrested at a G0-like cell cycle phase or checkpoint [7]. This quiescence may account for many treatment failures, hence, the only effective way to treat cancers generated by CSCs is to target the CSC population, blocking their ability to self-renew and generate multilineage differentiation [8], which provides a hierarchical organization of cells [9]. This hierarchy includes CSCs that produce committed progenitor cells which in turn, produce rapidly proliferating cells, finally resulting in the generation of fully differentiated cells [9]. Prevailing explanations for observed tumor cell heterogeneity are based on the influence of microenvironments and genomic instability, which prevent accurate replication and transmission of stable genotypes and phenotypes [5]. Several studies have proposed the existence of a functional microenvironment that supports CSCs, which is called the CSC niche. This microenvironment is composed of specialized endothelial cells, associated cells of mesenchymal origin, and extracellular matrix components. Signals that originate from this tumor niche regulate CSCs self-renewal, survival and ability to invade tissues. Scientific evidence suggests that CSCs have a role in all phases of tumorigenesis: initiation, progression, invasion and metastasis spreading [6]. Metastases develop when distant organs are seeded with CSCs that arise from a primary tumor; this implicates CSCs in the seeding and growth of metastatic lesions [6]. Table 1 summarizes the cellular abilities of tissue-specific SCs in comparison with CSCs.

The ideal cancer chemopreventive agent would selectively target CSCs together with the proliferating bulk of the tumor cells. As evidenced by many epidemiological studies [10], diet derived phytochemicals could act in tumor prevention and therapy. Molecules such as those derived from broccoli have successfully passed phase I clinical trials [11]. Many of these phytochemicals have been used in combination with conventional chemical drugs for tumor treatment [12]. In several cases, whole vegetable extracts were used and were shown to be able to inhibit proliferation and induce apoptosis in CSCs better than single phytochemicals [13].

In this review, we highlight the remarkable ability of phytochemicals to decrease the replicative capacity of CSCs, we describe the signaling pathways implicated in the phytochemical mediated targeting of those stem cells and cocktails of phytochemicals able to destroy CSCs.

**Table 1:** Cellular capacities of tissue-specific stem cells (SCs) and cancer stem cells (CSCs).

Cellular Capacities	Stem Cells (SCs)	Cancer Stem Cells (CSCs)	Reference
Evade apoptosis	NO	YES	[14]
Self-sufficiency in growth signals	NO	YES	[2]
Insensitivity to anti-growth signals	NO	YES	[2]
Tissue invasion and metastasis	NO	YES	[2]
Contact inhibition *	YES	NO	[2]
Sustained angiogenesis	NO	YES	[2]
Deregulation of cellular energetics	NO	YES	[2]
Avoidance of immune destruction	NO	YES	[2]
Genome instability and mutations	NO	YES	[2]
Tumor-promoting inflammation state	NO	YES	[2]
Self-renewal	YES	YES	[14]
Quiescence in G0-like phase	YES	YES	[3]
Anticancer drug resistance **	NO	YES	[3]

* Cancer stem cells continue to proliferate even if they come into contact with other cells; ** Cancer stem cells show high resistance to conventional anticancer drugs.

## 2. Isolation of Cancer Stem Cells (CSCs) from Tumors

CSCs have been identified in several cancer types, including various forms of leukemias as well as solid tumors through the detection of various surface antigens [15]. It has been demonstrated that brain, colon, prostate and lung CSCs bear the antigen CD133, while ovarian CSCs are CD24^+^ and CD133^+^, acute myeloid leukemia stem cells are CD34^+^ and CD38^−^, head and neck CSCs are CD44^+^, B-precursor acute Lymphocytic leukemia CSCs are CD34^+^, CD38^+^ and CD19^+^ [8]. Moreover, several *in vitro* assays have been used to identify stem cells, including sphere assays, serial colony-forming unit (CFU) assays and label-retention assays coupled with measurement of Aldehyde dehydrogenase 1 (ALDH1) activity [5]. ALDH1 is widely used as a marker to identify and isolate normal and cancer stem cells. Nowadays, the “gold standard” for assessing the activity of ALDH in viable cells consists of the use of flow cytometry and fluorescent substrates in the Aldefluor Assay [8]. In this method, cells expressing ALDH1 take up uncharged ALDH substrate Boron-dipyrromethene (BODIPY) amino-acetaldehyde (BAAA) by passive diffusion and then convert BAAA into negatively-charged BODIPY aminoacetate (BAA^−^). BAA^−^ is then retained inside cells, causing the subset of cells with a high ALDH activity (high ALDH) to become highly fluorescent. These high ALDH populations can be distinguished easily and specifically via comparison to the fluorescence expressed in the presence of the specific inhibitor of ALDH1 *N*,*N*′-diethylaminobenzaldehyde [16,17,18]. The availability of additional biomarkers for cancer stem cells, when combined with ALDH1 in a multimarker test may provide a way to improve specificity for clinical use [8].

## 3. Molecular Mechanisms of Self-Renewal

**Figure 1:**
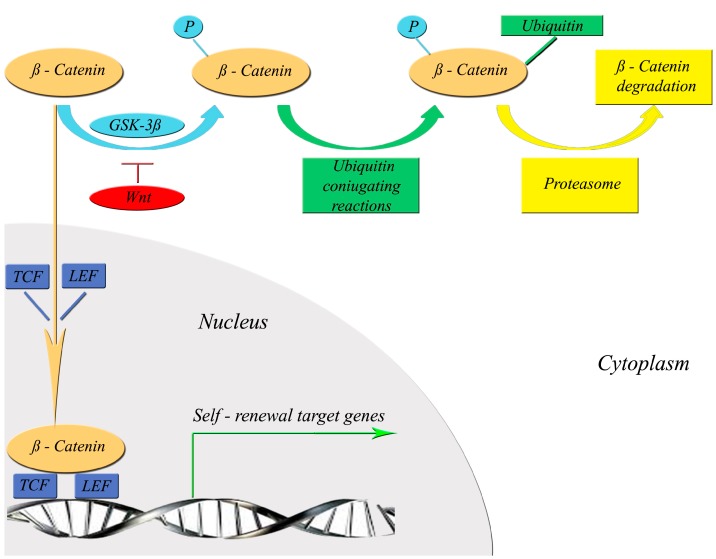
Signaling network of Wnt/β-catenin regulating the expression of self-renewal genes in cancer stem cells. In the presence of Wnt, GSK-3β is inhibited and β-catenin can translocate from the cytoplasm into the nucleus with consequent activation of self-renewal target gene transcription. Abbreviations: GSK-3β, glycogensynthasekinase-3β; TCF, T cell factor; LEF, lymphoid enhancer factor; Wnt, wingless-related integration site.

A number of studies have been conducted to find the genetic signatures that determine CSCs’ self-renewal [19]. Several genes and signaling pathways have been shown to have important regulatory functions for normal and cancer stem cells [19]. The following genes and signaling pathways are the most important: Hedgehog, a glycoprotein family involved in the pro-survival pathways [19]; Notch, a transmembrane receptor involved in the self-renewal processes [19]; Wnt/β-catenin, a family of secreted proteins involved in the self-renewal pathways [19] and Bmi-1, a transcriptional repressor factor with a role in the self-renewal processes and regulation of telomerase expression [19]. The most interesting candidate to be targeted with phytochemicals is the Wnt/β-catenin pathway, implicated in the pathogenesis of several cancers [20]. It has been shown that, in the absence of Wnt signaling, β-catenin remains in the cytoplasm, where it forms a complex with glycogen synthase kinase GSK-3β [21], able to phosphorylate β-catenin, which undergoes degradation. When the Wnt pathway is activated, GSK-3β is inhibited, blocking β-catenin phosphorylation. Unphosphorylated β-catenin is stable and translocates to the nucleus, where it binds to and activates the transcription factors T cell factor-lymphoid enhancer factor (TCF-LEF), which in turn increase the self-renewal and proliferation of CSCs [20,21]. Figure 1 shows in detail how the Wnt/β-catenin pathway regulates the expression of self-renewal genes in CSCs.

## 4. Phytochemicals Able to Kill Cancer Cells

Among chemopreventive dietary agents, the following are the most effective in reducing the proliferative activity of cancer cell lines: tea polyphenol epigallocatechin-3-gallate (EGCG), curcumin, resveratrol, lycopene, pomegranate extracts, luteolin, genistein, piperin, β-carotene and sulforaphane. These phytochemicals have been thoroughly studied for at least three decades. Indeed, research on phytochemicals began even before the potential role of CSCs in tumor development and propagation was known. However, these studies provide a huge body of knowledge, which can now be applied in the development of treatments against CSCs. Examples of these compounds are reported below.

EGCG is the most abundant polyphenol in green tea. It is able to induce the caspase 8 dependent apoptosis in tumor cell cultures and animal models [22,23]. There are several ongoing clinical trials involving EGCG alone or in combination with cisplatin and oxaliplatin because of EGCG’s ability to synergistically increase the efficacy of these conventional drugs against prostate carcinoma and colorectal cancer [24,25].

Curcumin, isolated from the rhizomes of the plant *Curcuma longa*, is the most important yellow pigment present in turmeric, a popular spice. Curcumin has been shown to interrupt the carcinogenetic process by inhibiting the initiation step or suppressing the promotion and progression stages in animal models [26,27]. Curcumin has also been reported to exhibit synergistic chemopreventive effects with other diet-derived polyphenols, such as genistein [28], EGCG [29] and embelin [30] and to increase the efficacy of many anticancer drugs including 5-fluorouracil [31,32], vinca alkaloid, vinorelbine [33], cisplatin and gemcitabine [34,35,36].

Resveratrol is a phytoalexin, an important constituent of red wine, abundant in the grape skin. Prophylactic use of resveratrol has been shown to reduce the number and size of esophageal, intestinal and colon tumors [37,38]. Resveratrol has been reported to prevent the development of 7,12-dimethylbenz(a)anthracene induced mammary carcinogenesis, and inhibit the growth of M.D. Anderson-metastatic breast 231 (MDA-MB231) tumor cell line xenografts. Moreover, it is thought to induce apoptosis in prostate cancer cell lines PC-3, DU145 and LNCaP and suppress the progression of prostate cancer in transgenic mice [39,40,41,42,43,44].

Lycopene is a natural antioxidant that gives tomatoes, watermelon, and pink grapefruit their red color. Epidemiological studies have shown that high intake of lycopene-containing vegetables is inversely associated with the incidence of certain types of cancer, including cancer of the digestive tract, prostate and cervix [45,46,47,48,49]. A combination of vitamin E, selenium, and lycopene has been shown to dramatically inhibit prostate cancer development and to increase disease-free survival [50]. Lycopene has also been shown to suppress the growth of lung cancer cells [51].

Luteolin is a flavonoid abundant in several green vegetables, such as cabbage, spinach and peppers. It exhibits anticancer effects [52] by inducing cell cycle arrest, senescence or apoptosis in cells of oral squamous cancer [53], human esophageal adenocarcinoma [54], lung carcinoma [55], human colon cancer [56], human hepatoma [57] and prostate cancer [58]. Indeed, Luteolin has been found to increase the efficacy of cisplatin against gastric cancer cells [59] and also of gemcitabine against pancreatic cancer [60].

Genistein is a phytoestrogen abundant in soybeans and soy products. Its consumption has been shown to be inversely correlated with the risk of prostate [61], breast [62,63] and endometrial [64] cancers. Moreover, genistein increased the antitumor activity of cisplatin in Mucin producer pancreatic cancer-3 (BxPC-3) tumor xenografts [65] and enhanced also the antitumor activity of All-Trans Retinoic Acid (ATRA) in lung adenocarcinoma cell therapy [66].

In addition to the aforementioned dietary agents, other natural compounds have been investigated for their chemopreventive potential. These include ellagic acid, lupeol, betulinic acid, ginsenosides, oleanolic acid, ginkolide B, and the pomegranate constituents cyanidin, delphinidin, and petunidin [67].

Sulforaphane is a potent chemopreventive compound found in Brassica vegetables, which are considered very healthy thanks to their high glucosinolate content. Sulforaphane derives from glucosinolate and glucoraphanin, after the hydrolysis performed by the enzyme myrosinase. In the human diet, only the *R*-isomer of sulforaphane is available; the *S*-isomer can only be obtained by chemical synthesis, which provides both isomers in variable proportions. A study was conducted on rat liver and lung cells, comparing the ability of the sulforaphane isomers R and S in modulating the detoxifying enzymes, quinone reductases and glutathione *S*-transferase [68]. It was found that the *R*-isomer was far more effective in upregulating quinone reductases and glutathione *S*-transferase activies and protein levels compared to the *S*-isomer. This finding highlights the clear superiority of the *R*-isomer of sulforaphane as a chemopreventive compound [68].

Sulphoraphane has also been tested in men with recurrent prostate cancer and has recently entered phase II clinical trials [69]. Patients receiving sulforaphane-rich broccoli extracts, showed a smaller increase in prostate specific antigen (PSA), compared to untreated patients [69].

Several other mixtures of flavonoids purified from vegetables [23,70] or secoiridoids purified from virgin olive oil extracts [71] have been used to inhibit cancerogenesis. Fabiani *et al.* [71] demonstrated that a virgin olive oil phenol extract (PE) inhibited proliferation and induced apoptosis in the human promyelocytic cell line HL60. Phenolic compounds of virgin olive oil have also been used to inhibit the spreading of metastases originating from the colon cancer cells HT115, both *in vitro* and *in vivo* [72]. The decrease in HT115 invasion was associated with a statistically significant reduction in integrins which are connected to several signaling pathways [73].

Numerous cell-signaling pathways are activated by dietary phytochemicals and the same compound may activate different pathways, depending on the cell types. The optimal condition is attained when the phytochemical increases the levels of the pro-apoptotic p53 and decreases the levels of the main pro-survival factors: Epidermal growth factor receptor (EGFR), nuclear factor-kappa B (NF-κB), activator protein 1, Signal transducer and activator of transcription (STAT), survivin, metalloproteinases 2 and 9, Vascular endothelial growth factor (VEGF), B-cell leukemia/lymphoma 2 (Bcl-2) [74].

## 5. Phytochemicals that Selectively Kill Cancer Stem Cells

From the huge number of studies, focusing on phytochemicals exhibiting cytotoxic effects on most tumor cells, it emerged that some of these phytochemicals are also able to kill CSCs. The cytotoxicity very often followed a hormetic mechanism, in which a specific chemical compound induces opposite effects at different doses; most commonly, there is a beneficial effect at low doses and a toxic effect at high doses [75].

One phytochemical that follows a hormetic mechanism is sulforaphane. Zanichelli *et al.* [75] suggest that the isomer *R*-sulforaphane (*R*-SFN) may be considered a hormetic dietary supplement; in fact, low doses of *R*-SFN promote human mesenchimal stem cell (MSC) proliferation and protect them from apoptosis and senescence, while high doses show a cytotoxic effect, causing the induction of cell cycle arrest, apoptosis and senescence. The beneficial effects of *R*-SFN can be attributed to its antioxidant properties, while its cytotoxic effects can be ascribed to pro-oxidant properties stemming from its ability to cause radical oxygen species (ROS) production and Glutathione in its reduced form (GSH) depletion [75]. Since the sulforaphane is able to interfere with the physiology of stem cells, researchers have begun using this compound and other phytochemicals with the aim of killing CSCs.

A very recent study showed the effectiveness of sulforaphane extracted from broccoli in inducing apoptosis in pancreatic CSCs by interfering with NF-κB anti-apoptotic signaling [76]. Daily injection of sulforaphane for two weeks suppressed tumor growth in primary Nonobese diabetic/Severe combined immunodeficient (NOD/SCID) mice and reduced ALDH-positive cell population in the tumors [77]. More importantly, it was found that the tumor cells derived from sulforaphane-treated mice were not able to form secondary tumors in recipient mice for up to 33 days. These data are consistent with the *in vitro* observation that sulforaphane preferentially targeted cancer stem/progenitor cells instead of the bulk cell population and that the preference of sulforaphane for killing CSCs may be significant for chemoprevention. Sulforaphane was able to downregulate the Wnt/β-catenin self-renewal pathway in breast cancer cells by inducing β-catenin phosphorylation, leading to its degradation by the proteasome, through the activation of GSK-3β [76]. Sulforaphane was able to target breast CSCs as shown by the mammosphere formation assay, Aldefluor assay, and tumor growth upon reimplantation in secondary mice [78].

It has also been shown that curcumin and piperine have the characteristics to affect CSCs, as documented by decreased mammosphere formation in the fraction of cells expressing the ALDH1 marker [79]. In contrast, these compounds have little or no effect on differentiated cells [80,81].

Curcumin is able to inhibit Wnt signaling pathway in MCF7 cells, as shown by the TCF-LEF reporter assay system [79]. Piperine is also able to inhibit breast CSCs self-renewal by targeting Wnt signaling [79]. Piperine may increase curcumin’s effects by inhibiting curcumin’s efflux via P-glycoprotein (ATP-binding cassette sub-family B member 1 (ABCB1) or Multi drug resistance 1 (MDR1)) efflux pump [82,83,84] and by downregulating NF-κB release [85].

β-Carotene has been identified as a phytochemical able to inhibit the growth of CSCs in neuroblastoma [86]. Neuroblastoma is the most common malignant tumor of the neural crest and arises within the sympathetic nervous system [86]. Treatment with β-carotene has been found to induce the differentiation of neuroblastoma cells and to decrease the self-renewal characteristics of CSCs [78], thereby preventing recurrence and metastasis. Lee *et al.* demonstrated that β-carotene can enhance the cytotoxic effects of cisplatin against neuroblastoma CSCs and that β-carotene exhibits excellent anti-CSC qualities. Hence, it has been suggested that β-carotene in synergy with cisplatin may represent a potent medical adjunct for neuroblastoma treatment.

An interesting paper was recently published on the ability of *Sasa quelpaertensis* extract (SQE) to exhibit toxicity on colon HCT116 and HT29 CSCs [13]. Both cell lines were CD133^+^ and CD44^+^ double-labeled and when injected subcutaneously into nude mice, all the animals developed tumors. SQE supplementation (300 mg/kg body weight) was found to weakly inhibit colon tumor growth, yet it significantly suppressed the expression of CSC markers, Wnt/β-catenin signaling, and hypoxia inducible factor-1α (HIF-1α) signaling in the xenografts. SQE contains various polyphenols, including p-Coumaric Acid and tricin, which regulate the metabolic activation of potential carcinogens and are recognized by xenobiotic metabolizing enzymes [87]. Table 2 summarizes the phytochemicals or extracts able to kill CSCs indicating the molecular mechanism that is involved in their action and the cell marker that demonstrates their effect.

**Table 2:** Phytochemicals that are able to kill the cancer stem cells of tumors through specific molecular mechanisms.

Phytochemicals or Extracts	CSCs Type	Molecular Mechanism	Reference
EGCG	Breast cancer	Inhibits Wnt signaling	[88]
Piperine	Breast cancer	Inhibits Wnt signaling	[79]
Sulforaphane	Breast cancer	Decreases ALDH1 activity	[77]
		Inhibits Wnt signaling	
	Pancreatic cancer	Induces apoptosis, activating caspase 3	[89]
		Downregulates β-catenin	
β-Carotene	Neuroblastoma	Inhibits Wnt signaling	[86]
		Induces CSC differentiation	
Quercetin	Pancreatic cancer	Inhibits Wnt signaling	[90]
Resveratrol	Pancreatic cancer	Induces apoptosis, activating caspase 3	[91]
	Colorectal cancer	Inhibits Wnt signaling	[92]
Genistein	Pancreatic cancer	Decreases number of mammospheres	[92]
		Decrease number of CD44^+^ cells	
Curcumin	Breast cancer	Decreases number of mammospheres	[79]
		Decreases ALDH1 activity	
	Colon cancer	Decreases ALDH1 activity	[93]
		Decreases number of CD44^+^, CD133^+^, CD166^+^ cells	
		Induces apoptosis	
	Colorectal cancer	Induces G2/M phase arrest	[94]
		Downregulates β-catenin	
	Prostate cancer	Induces G2/M phase arrest	[95]
		Inhibits Wnt signaling	
*Sasa quelpaertensis* extract	Colon cancer	Induces CSC differentiation	[13]
		Inhibits Wnt signaling	

## 6. Conclusions

Single phytochemicals and enriched natural extracts able to interfere with self-renewal and drug resistance pathways in CSCs have been identified. This is a milestone in the improvement of cancer treatment because the synthetic anticancer drugs that are currently used are often highly toxic for healthy organs and weakens the patient’s immune system. These phytochemical compounds or extracts, which show low levels of toxicity for normal cells can be used against cancers in combination with other phytochemicals, yielding powerful synergistic effects. The main obstacle to overcome consists in finding a way to combine the single drugs or extracts into very active cocktails of phytochemicals able to cope with molecular targets in the signaling network of CSCs sustained cancerogenesis in several tumors. Moreover, it is necessary to compare the anticancer effects of natural phytochemicals extracted from vegetables with synthetic products that may be less efficient than the natural forms due to different mixtures of stereoisomers. Finally, it is imperative to gain a better understanding of the signaling pathways that govern the self-renewal and survival of CSCs. Current findings on phytochemicals warrant further investigation in order to better define the role played by these molecules in human cancer therapy.

## Abbreviations

ABCB1: ATP-binding cassette sub-family B member 1; ALDH1: Aldehyde dehydrogenase 1; BAA^−^: BODIPY aminoacetate; BAAA: BODIPY amino-acetaldehyde; Bmi-1: B lymphoma Mo-MLV insertion region 1; Bcl-2: B-cell leukemia/lymphoma 2; CD: Clusters of Differentiation; CFU: Colony-forming unit; CSC: Cancer stem cells; EGCG: Epigallocatechin-3-gallate; EGFR: Epidermal growth factor receptor; GSH: Glutathione in reduced form; GSK-3β: Glycogen synthase kinase-3β; HIF-1α: Hypoxia inducible factor-1α; LEF: Lymphoid enhancer factor; MDR1: Multi drug resistance 1; MSC: Mesenchymal stem cells; NF-κB: Nuclear factor-kappa B; NOD/SCID: Nonobese diabetic/severe combined immunodeficient; PE: Virgin olive oil phenolic extract; PSA: Prostate specific antigen; ROS: Radical oxygen species; *R*-SFN: *R*-isomer of sulforaphane; SC: tissue-specific stem cells; SQE: *Sasa quelpaertensis* extract; STAT: Signal transducer and activator of transcription; TCF: T Cell factor; Wnt: Wingless-related integration site.
